# Results in treatment of distal femur fractures using polyaxial locking plate

**DOI:** 10.1007/s11751-013-0182-7

**Published:** 2013-12-21

**Authors:** R. Pascarella, C. Bettuzzi, G. Bosco, D. Leonetti, S. Dessì, P. Forte, L. Amendola

**Affiliations:** Department of Orthopaedics and Traumatology, Maggiore Hospital, Bologna, Italy

**Keywords:** Distal femur, Fracture fixation, Polyaxial locked plate, Clinical outcome, Radiographic outcome

## Abstract

Indications and techniques of locked plate fixation for the treatment of challenging fractures continue to evolve. As design variant of classic locked plates, the polyaxial locked plate has the ability to alter the screw angle and thereby, enhance fracture fixation. The aim of this observational study was to evaluate clinical and radiographic results in 89 patients with 90 fractures of the distal femur treated, between June 2006 and November 2011, with such a polyaxial locked plating system (Polyax™ Locked Plating System, DePuy, Warsaw, IN, USA). Seventy-seven fractures formed the report of this study. These cases were followed up until complete fracture healing or for a mean time of 77 weeks. At the time of last follow-up, 58 of 77 fractures (75.3 %) progressed to union without complication and radiographic healing occurred at a mean time of 16.3 weeks. Complications occurred in ten fractures that did not affect the healing and in nine fractures that showed delayed or non-union. The mean American Knee Society Score at the time of final follow-up was 83 for the Knee Score and 71.1 for the Functional Score. In conclusion, there is a high union rate for complex distal femoral fractures associated with a good clinical outcome in this series.

## Introduction

Angle-stable locked plates have been used successfully for distal femoral fractures where the new design imparts a higher degree of stability and provides better protection against primary and secondary losses of reduction [1–9]. First-generation locked plates had fixed-angle threaded holes allowing a stable periarticular fixation but with all holes in the plate holding the screws at the same angle. This fixed angle was disadvantageous for some types of fracture of the distal femur, e.g. in screw placement around prostheses in periprosthetic fractures [10–13].

To minimise these problems, different polyaxial locking plates have been introduced, extending the concept of polyaxial screws of spinal fixation systems [14]. These plates allow many options in screw angulation enhancing the possibility of fracture fixation. This versatility allows for a stable osteosynthesis of highly comminuted or osteoporotic fractures. Several technologies exist to obtain variable axis locking such as self-locking bushings or screws with two components where a cap locks the screw in a chosen direction.

An important issue is the reliability of the polyaxial plate in biomechanical performance. Recent studies [15] reveal no significant difference when compared with traditional plates with no failures in the interface between screw and plate when different polyaxial plates were analysed. There are a few reports [12] of clinical experience with polyaxial implants, and early observation confirms good performance and complication rates similar to first-generation fixed-angle plates.

The aim of this study was to assess the results of a series of distal femur fractures treated at our institution with the Polyax™ Locked Plating System.

## Materials and methods

Between June 2006 and November 2011, 89 patients with a total of 90 fractures of the distal femur were included in this observational study and treated with open reduction and internal fixation with the Polyax™ Locked Plating System (DePuy, Warsaw, IN, USA). All clinical and radiological data were recorded from the beginning of the study period in a specifically built database. All patients gave their informed consent for surgery, and the study was authorised by the local ethical committee. The study was performed in accordance with the ethical standards of the 1964 Declaration of Helsinki as revised in 2000.

The group included 35 male and 54 female patients. The mean age at the time of the fracture was 62 years (range 16–96). Anteroposterior and lateral view radiographs of the knee were obtained to establish the fracture pattern, classification and pre-operative planning. The distal femoral fractures were classified according to the AO system: there were five type 32A1, one type 32A3, four type 32B1, two type 32C1, two type 32C3, ten type 33A1, eight type 33A2, twelve type 33A3, one type 33B1, three type 33B2, one type 33B3, four type 33C1, twenty-eight type 33C2 and nine 33C3.

Twenty-six (29.2 %) were polytrauma patients (ISS > 17). Periprosthetic femur fractures occurred in 13 cases (9 hips and 4 knees), and four patients presented with a fracture after intramedullary nailing of the proximal femur. There were 67 closed (74.4 %) and 23 open fractures (25.5 %): nine type 1, eight type 2 and six type 3 according to Gustilo and Anderson classification [16]. Open fractures were treated with intravenous antibiotics, tetanus prophylaxis, emergency debridement and external fixation or plating according to Gustilo soft-tissue injury type within 6–8 h.

The timing of definitive surgery depended on the soft-tissue conditions. Eighteen patients with severe soft-tissue swelling and skin blistering were treated with temporary short-term external fixation and then underwent delayed open reduction and internal fixation. This staged procedure was performed in four cases of closed fractures for multiply injured patients and in 14 cases of open fractures (two type 1, six type 2, six type 3 according to Gustilo and Anderson classification) [16]. The mean time of delay for open reduction and fixation was 21 days (range 7–45 days).

The surgical procedure was performed by the same team of experienced surgeons. In 19 cases with bone loss, a synthetic (4 cases) or homograft (15 cases) was used. An allograft cortical strut was used in 11 fractures in order to improve stability on the medial side of the metaphyseal bone loss.

Routine post-operative radiographs were performed and analysed. Malalignment was defined as the presence of more than five degrees of angulation in any plane.

All patients had physical (compression stockings, calf or foot pumps) and pharmacologic prophylaxis for prevention of venous thromboembolism. Post-operative rehabilitation consisted of isometric quadriceps strengthening, continuous passive motion of the knee and ambulation with crutches and no weight bearing for 8–10 weeks. Then, gradual weight bearing was allowed when there was evidence of progressing union on X-rays.

All patients were routinely followed in the outpatient clinic; radiological and clinical examinations (1, 2, 3, 6 months after surgery and then yearly) were conducted and the following noted: time to union, loss of reduction, hardware failure (loosening or breakage) and local or systemic complications.

Union was defined as bridging of three of the four cortices and disappearance of the fracture line on the plain radiographs for a patient who was able to bear full weight [17]. There are several published definitions of non-union, but none is universally accepted. According to Rodriguez-Merchan [18], we define non-union as a fracture that did not heal within 8 months and required second surgery. To assess loss of reduction and hardware failure, the radiographs at the latest follow-up were compared with the first post-operative ones. Patients were assessed using the Knee Society clinical rating system subdivided into a Knee Score (AKSS) based on three main clinical parameters (pain, joint stability and range of movement) and a Functional Scores (AKFS) based on the patient’s perception of general knee function in specific activities (walking ability and ascending/descending stairs) [19]. This dual rating system eliminates the problem of declining Knee Scores associated with increasing age or other medical conditions. A score between 85 and 100 points is considered excellent; 70–84, good; 60–69, fair; and <60, poor.

## Results

Six patients were lost to follow-up prior to fracture union (four of them were foreigners who returned to their countries), four elderly patients (age >85 years) died for other medical problems before fracture union and three polytrauma patients died within 1 month after surgery from multi-organ failure. Therefore, 76 patients (with 77 fractures) were included in this report. They were followed up until complete fracture healing and for a minimum of 24 weeks (range 24–230) with mean follow-up of 77 weeks. At the time of last follow-up, 58 of 77 fractures (75.3 %) united without complication (Table 1, group a): radiographic healing occurred at a mean time of 16.3 weeks (range 12–24 weeks). Only one intraoperative complication occurred in our series (1.3 %) which did not lead to a delayed union (Table 1, group b); during fracture reduction, a dislocation of the nearby knee prosthesis increased surgery time and required post-operative immobilization. Eight patients with nine fractures (11.7 %) also had post-operative complications that had effect on healing (Table 1, group c).

**Table 1:** Summary of the results

Group	No of fracture (%)	Complication
(a) Union without complication	58 (75.3 %)	
(b) Union with intraoperative complication	1 (1.3 %)	1 Knee prosthesis dislocation
(c) Union with late post-operative complication	9 (11.7 %)	2 Bone deformity/stiff knee 5 Soft-tissue irritation 2 Stiff knee
(d) Complications of bone healing	9 (11.7 %)	1 Deep infection 1 Fracture 7 Non-union (9.1 %)

One of these patients had mental health illness and had sustained bilateral distal femur fractures from self-injury. This was treated with temporary external fixation followed by open reduction and internal fixation. Ten weeks after surgery, complete loosening of the hardware was observed in the right femur due to plate malpositioning and required implant removal. It was then managed by cast immobilization for 6 weeks. The fractures finally healed with a significant bone deformity on the right side and stiff knees in both lower limbs. This bedridden patient did not require other surgical procedures.

Five patients who reported pain and tissue irritation after fracture union underwent hardware removal with a corresponding decrease in symptoms except for one case with patello-femoral joint pain. Two patients underwent arthrolysis and quadricepsplasty for stiff knee problems after which a final range of motion was recorded as complete extension to 90 degrees of knee flexion.

Nine fractures (11.7 %) developed complications of bone healing (Table 1, group d): one deep infection, one fracture near the plate and seven with aseptic non-union.

One deep infection developed in our series despite a high percentage of open and high-energy injuries. This 76-year-old patient with a closed fracture (33-C2), affected by insulin-dependent diabetes and a severe peripheral neuropathy, had an early post-operative deep infection and opted for an above-the-knee amputation instead of limb salvage.

**Fig. 1:**
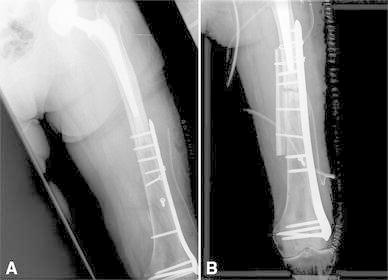
Female 86 years. **a** Insufficient plate length to fix a distal femur fracture in a patient with a hip prosthesis. **b** Fracture occurred between the stem and plate; this was treated by a longer plate and cortical strut graft

Another patient with a hip prosthesis presented a periprosthetic fracture because of a plate of insufficient length was used to fix the fracture. He underwent revision surgery with a longer plate and a cortical strut graft to augment fixation (Fig. 1a, b).

**Fig. 2:**
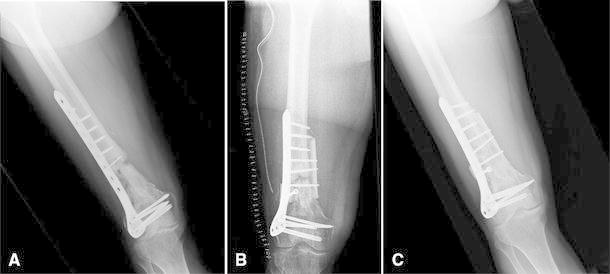
Male 46 years. **a** Non-union in a complex distal femur fracture. **b** Post-operative X-ray after revision surgery using homoplastic cortical strut and a new plate. **c** 1.5 years after second surgery with the AP view radiograph showing fracture healing

Non-union (Fig. 2a) occurred in seven patients (9.1 %). Four patients were treated at 36 weeks after the first surgery with homoplastic cortical bone graft opposite to a new plate (Fig. 2b). In all cases, the first implant was well fixed at the time of removal with no evidence of screw loosening, toggle or loss of distal fixation. Three of these patients healed after about 4 months after revision surgery (Fig. 2c). The fourth patient, a heavy smoker, underwent further surgery for persistence of non-union.

**Fig. 3:**
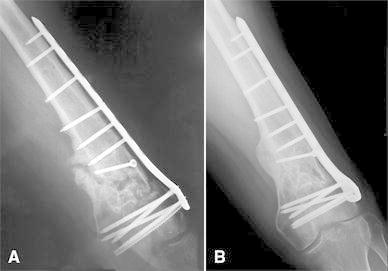
Male 46 years. **a** AP view before second surgery shows a non-union. **b** Radiograph at last follow-up shows fracture healing

Two patients were treated with cancellous autograft 32 weeks after the first surgery. Both cases healed at about 3.5 months after second surgery (Fig. 3a, b). Another patient, in whom post-operative X-rays showed plate malpositioning, had with symptomatic severe knee pain. Subsequent image studies indicated insufficient healing progression with bone deformity. The clinical evaluation suggested a lesion of medial collateral ligament. This patient underwent conversion to an arthroplasty with a rotating hinge prosthesis about 6 months later.

Excluding the nine patients who did not heal after first surgery and the patient with the history of mental health illness, the remaining 66 patients were assessed for outcome using the Knee Society clinical rating system and with X-rays at a mean follow-up of 77 weeks. The mean Knee Score at the time of the latest follow-up was 83 points (range 57–100), and the mean Functional Score was 71.1 points (range 20–100).

Being an observational study, it was not possible to determine pre-operative or other factors linked to healing and clinical outcome; this was due to the large number of variables examined about patient injury and treatment leading to a very heterogeneous sample.

Eleven of these 66 fractures (16.7 %) had femoral malalignment of <10° (especially in varus) at post-operatively X-ray. At last follow-up, no loss reduction and fair clinical results were reported except in one patient with knee pain who underwent removal of hardware 13 months after surgery.

## Discussion

Distal femoral fractures are challenging injuries despite improvements of fixation techniques and plate designs. Some authors [20, 21] have demonstrated the ability of locked plates to absorb more energy before failure compared with angled blade plates or retrograde intramedullary nails, thereby having a lower incidence of loss of fixation. Although no agreement exists on management of complex distal femoral fractures, the results reported by several authors [1, 2, 4–6, 8, 11, 22] suggest modern locking plates represent an advance for fixing different fracture patterns in this region. These include either high-energy fractures with severe bone comminution that may be further complicated through open injury, fractures in older people with poor bone quality and periprosthetic fractures.

The disadvantages of first-generation locked plates include the uniaxial screw trajectories. These screws trajectories cannot account for differences in femoral anatomy, fracture patterns or variations in plate positioning. In recent studies, biomechanical characteristics of a polyaxial system were analysed in comparison with uniaxial first-generation locking plates [15]. Despite the large forces applied, there were no failures of the polyaxial screw–plate interface and screw angle did not reduce the overall strength of these constructs, hence lending support to the biomechanical effectiveness of polyaxial plate designs under axial loading.

This observational study reports the experience with the Polyax™ Locked Plating System (DePuy, Warsaw, IN, USA) for treatment of supracondylar femoral fractures. Intraoperative advantages with variable axis screws [12, 23] include the possibility to reduce the effects of obstacles to adequate periarticular fixation. Such devices allow maximal periarticular fragment fixation through use of multiple screws or by the option to spread screws in a remote segment.

Clinical experience with this new type of locking construct is not widespread, and only a reports are available in the literature [12, 22]. Haidukewych et al. [12] reported a series of 56 periarticular knee fractures (including only 25 in the distal femur) treated by using the Polyax plate; fracture healing was achieved in 94 % of the cases with satisfactory clinical outcomes for most of patients. Other previously published studies of fractures of the distal femur treated with different kinds of locked plate [6, 8, 24–29] also demonstrated non-union rates similar to those in the current study (Table 2).

**Table 2:** Non-union of distal femur fractures treated with locking plates in different studies

Study	No. of fractures	% Non-unions
Fankhauser et al. [24]	30	0
Gaines et al. [25]	109	8
Kayali et al. [26]	27	0
Kregor et al. [6]	103	2
Markmiller et al. [27]	20	10
Schandelmaier et al. [8]	54	2
Schutz et al. [28]	52	4
Vallier et al. [29]	46	9
Current study	77	9.1

Our results are comparable to recent published series. This observational study has several limitations but most importantly is heterogeneity in the sample; there is a large number of differing variables in patient, injury characteristics and treatment pathways that make determination of factors that influence healing and clinical outcome difficult. There is, in addition, a lack of a control group.

## Conclusion

The results of this observational study indicate that a polyaxial locking plate offers clinical and radiographic outcomes similar to those treated with fixed-trajectory locking plates but with greater fixation versatility. The system provides a high degree of angular and axial stability in a series of complex distal femoral fractures.
